# Successful treatment of Becker's nevus with dermabrasion by sandpaper: A case report

**DOI:** 10.1002/ccr3.4725

**Published:** 2021-08-30

**Authors:** Hossein Hafezi, Hamid Galehdari, Mina Rezaie, Reza Moeini

**Affiliations:** ^1^ Department of Dermatology Isfahan University of Medical Sciences Isfahan Iran; ^2^ School of Medicine Isfahan University of Medical Sciences Isfahan Iran

**Keywords:** Becker nevus, dermabrasion, sandpaper

## Abstract

Lasers have been widely used for treatment of Becker nevus. Here, we report a case of Becker nevus which did not respond to laser therapy and was treated successfully by dermabrasion with sandpaper with no following complications.

## Graphical abstract

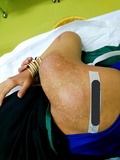

## 1 INTRODUCTION

Here, we report a case of Becker's nevus in a 19‐year‐old female patient who was initially treated with Q‐switched laser for several times, which was ineffective. We tried dermabrasion with sterilized sandpaper, which led to full recovery of the lesion without any further complications.

Becker Melanosis was first described by Samuel William Becker in 1948^1^ as unilateral melanosis and hypertrichosis and has been called Becker's nevus since then. Becker's nevus happens due to an overgrowth of the epidermis and can be congenital or acquired. The etiology and pathogenesis of Becker's nevus still remain uncertain; however, there is evidence that suggests the role of androgens in pathogenesis of Becker's nevus including male preponderance, peripubertal development, hypertrichosis, acneiform lesions within the patches, and an increase in androgen receptors.^2^, ^3^, ^4^, ^5^ Becker's nevus is often described as tan‐to‐brown skin lesions with hypertrichosis mostly located on the shoulder, the chest, or the back.^6^ Various therapeutic methods for Becker's nevus have been studied. Q‐switched lasers have shown acceptable efficacy in treating hyperpigmentation in Becker's nevus but are followed by a considerable chance of recurrence.^7^, ^8^ Here, we report a case of Becker's nevus in a 19‐year‐old female patient who was treated by dermabrasion following an unsuccessful laser therapy.

## 2 CASE PRESENTATION

**FIGURE 1:**
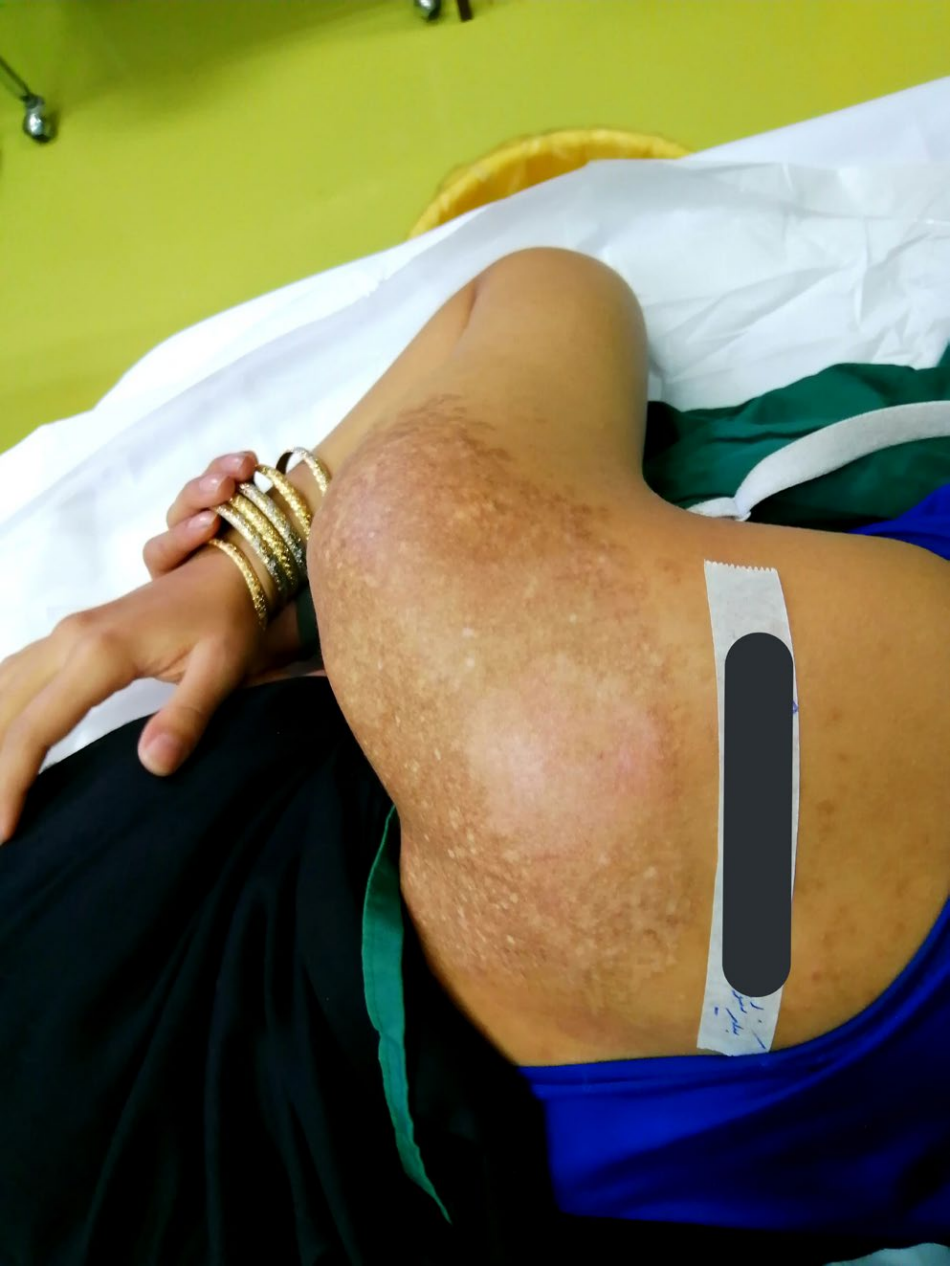
Becker nevus lesion on the shoulder

**FIGURE 2:**
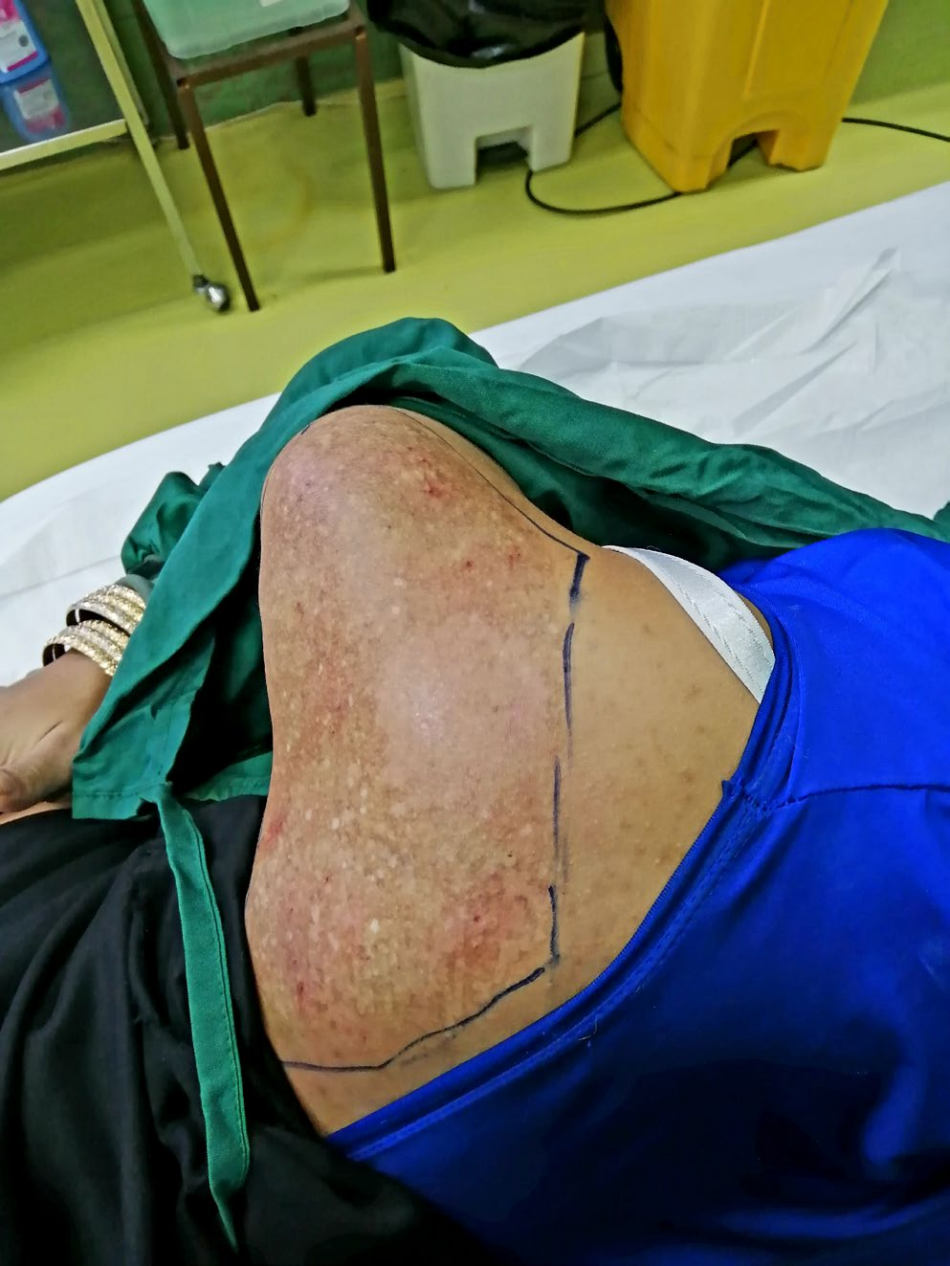
10 × 10 cm area that responded to previous dermabrasion

**FIGURE 3:**
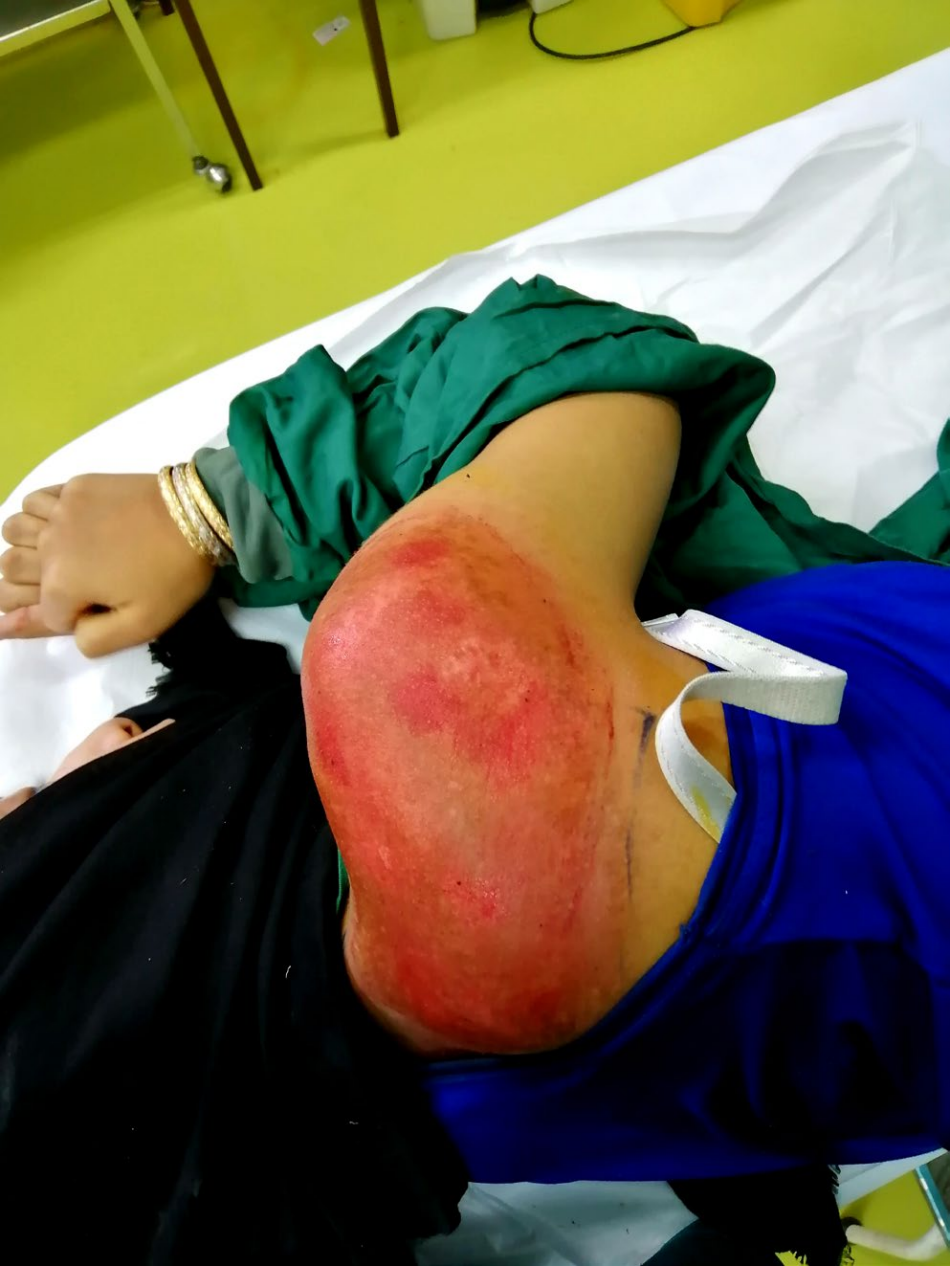
Dermabrasion of the whole lesion

The patient was an otherwise healthy 19‐year‐old female patient who presented to our dermatology department with the chief complaint of an irregular color change on her right shoulder and scapula since she was 15 years old. The lesion had developed in size over time. By the time she was referred to our dermatology department, the lesion was 20 × 20 cm (Figure 1). She did not complain of itching, bleeding, or scaling. She denied having any systemic diseases or using any drugs, and she did not have hypertrichosis. After examining the patient, she was diagnosed with Becker's nevus, which was later confirmed by biopsy. There were no findings to support the diagnosis of other conditions. Re‐evaluation of the patient by another dermatologist confirmed the diagnosis. We initiated laser therapy by Q‐switched ruby laser (694 nm, energy: 5 J, spot size: 3 × 3 mm), but there were no signs of improvement after three sessions. Since laser therapy was unsuccessful, we decided to use the dermabrasion method to remove the epidermis and upper dermis in the affected area by Becker's nevus. After injecting local anesthetics, we used a sterilized 180 grit sandpaper and removed epidermis and superficial dermis in a 10 × 10 cm surface manually in circular patterns. Prophylactic Cephalexin capsules and topical Mupirocin ointment were prescribed for the patient to use every 6 h for a week. We followed up the patient for 3 months afterward and the procedure was not followed by any complications including skin bleeding, infection, scarring or skin discoloration. The lesion was successfully removed after only one session of dermabrasion and did not recur afterward (Figure 2). After the improvement of the selected area of the lesion, we decided to use dermabrasion on the whole surface of the lesion using the same exact procedure. The patient was followed up for any complications, and the lesion was significantly improved after 3 months (Figure 3).

## 3 DISCUSSION

Becker's nevus is often characterized as an asymptomatic discoloration of the skin, which does not require primary treatment, but the cosmetic burden of hyperpigmentation or hypertrichosis in some patients makes the treatment necessary.

Different treatment modalities have been used to treat Becker's nevus including Q‐switched ruby laser (694 nm) with variable success.^9^, ^10^, ^11^, ^12^ Er:YAG laser has been found more effective than Q‐switched Nd:YAG system in a study by Trelles et al,^8^ and the results of this study have been supported by a small case series in Saudi Arabia.^13^ Taheri et al^14^ reported a successful treatment by topical solution of flutamide, which also supports the role of androgens in the pathogenesis of Becker's nevus. In this case, despite three sessions of laser therapy by Q‐switched ruby laser, there was no improvement so we decided to change our therapeutic method to dermabrasion by sandpaper.

Dermabrasion is a resurfacing technique, which removes the epidermis in order to promote re‐epithelialization. It is a simple mechanical method, which has been used to treat hyperpigmented skin lesions long before the laser systems were available. It is often performed by a motorized hand piece, which rotates on the skin and penetrates to the papillary dermis.^15^ Dermabrasion by sandpaper is an effective and inexpensive method that has been used for treating different types of skin conditions such as burn scars, acne scars, Hailey‐Hailey disease, and hypertrophic scars.^16^, ^17^ It is a sterilized procedure that can be performed under local or general anesthesia. It is the simplest technique for dermabrasion but it is difficult to perform in finer areas like face.

Before starting the dermabrasion procedure, patients should be examined and questioned about any bleeding disorders, immunosuppression, prior herpes simplex infection, keloidal or hypertrophic scarring, and prior isotretinoin therapy.^18^ Previous case reports have described delayed wound healing and keloid formation after dermabrasion in patients who were exposed to isotretinoin.^19^ Most frequent complications following dermabrasion are spot bleeding, erythema, milia formation, hypopigmentation, hyperpigmentation, and keloid formation. Hyperpigmentation is usually seen 4–6 weeks after the procedure, but it is mostly transient. Hyperpigmentation and hypopigmentation mostly affect the Fitzpatrick skin classification of IV and V.^20^ In our case, we followed the patient thoroughly after the procedure was performed. There were no complications including hypopigmentation, hyperpigmentation, and keloid formation despite the fact that the patient had Fitzpatrick IV skin type and that the lesion was located on her shoulder making her more susceptible to complications.

## 4 CONCLUSION

Dermabrasion by sandpaper is a cost‐effective technique for removing skin lesions and can be used as an effective and practical method for treating Becker's nevus.

## CONFLICT OF INTEREST

None declared.

## AUTHOR CONTRIBUTIONS

All the authors listed in the manuscript have participated actively in preparing the final version of this case report.

## ETHICAL APPROVAL

Written consent was taken from the patient for publishing the case report and viewing the images. This case report was approved by the bioethics committee of Isfahan University of Medical Sciences.

## Data Availability

No datasets were generated or analyzed during this case report.
